# Epigenetic modifications in developmental coordination disorder: association between DNA methylation and motor performance

**DOI:** 10.3389/fcell.2025.1647365

**Published:** 2025-09-09

**Authors:** Fangfang Huang, Huizhen Li, Haizhen You, Yuantao Su, Huijuan Peng, Wenchong Du, Jing Hua

**Affiliations:** ^1^ Department of Women’s and Children’s Health Care, Shanghai Key Laboratory of Maternal Fetal Medicine, Shanghai Institute of Maternal-Fetal Medicine and Gynecologic Oncology, Shanghai First Maternity and Infant Hospital, School of Medicine, Tongji University, Shanghai, China; ^2^ NTU Psychology, School of Social Sciences, Nottingham Trent University, Nottingham, United Kingdom

**Keywords:** developmental coordination disorder, DNA methylation, motor performance, neurodevelopment, epigenetics

## Abstract

**Objective:**

Developmental Coordination Disorder (DCD) is a common neurodevelopmental condition characterized by impaired motor coordination. However, the biological mechanisms underlying DCD remain largely unclear. This study aimed to investigate the potential role of DNA methylation in the pathogenesis of DCD.

**Methods:**

Genome-wide DNA methylation analysis was conducted using peripheral blood samples from children with and without DCD. Forty-two key differentially methylated probes (DMPs) were selected for targeted validation using MethylTarget™ sequencing.

**Results:**

A total of 416 DMPs were detected. Using the Bumphunter and ProbeLasso algorithms, 48 and 22 differentially methylated regions (DMRs) were identified, respectively. Among the key DMPs, methylation levels at cg18187326 (*FAM45A*) and cg11968956 (*FAM184A*) were significantly associated with both total motor and gross motor scores. In addition, cg03597174 (*SEZ6*) was negatively associated, while cg05986449 (*GPD2*) was positively associated with gross motor function.

**Conclusion:**

These findings provide preliminary evidence that specific DNA methylation alterations may influence early motor development and potentially contribute to the pathogenesis of DCD. DNA methylation markers may serve as novel biomarkers for early diagnosis and targeted intervention in children with DCD.

## Introduction

DCD is a neurodevelopmental disorder characterized by impaired motor coordination, with a current prevalence of approximately 5%–6% among children aged 5–11 years (Biotteau et al., 2020; Blank et al., 2019). Children with DCD often exhibit slow, clumsy, or inaccurate movements, which can significantly affect their daily activities such as writing and riding a bicycle (Hua et al., 2020). Beyond motor impairments, DCD is also strongly associated with lower cognitive function (Wilson et al., 2013), learning difficulties (Harrowell et al., 2018), and mental health problems (Lingam et al., 2012). Importantly, DCD often persists into adulthood, with approximately 30%–70% of individuals continuing to experience motor dysfunction, potentially leading to non-motor problems such as depression, anxiety, and low self-esteem (Biotteau et al., 2019; Losse et al., 1991). Given the long-term impact of DCD, it is essential to investigate the biological mechanisms of DCD to develop effective intervention strategies.

The mechanisms underlying DCD have not been clearly defined, but evidence suggests that they may involve the interaction of genetic and environmental factors. Epigenetic mechanisms, including DNA methylation, histone modifications, and non-coding RNA regulation, are key mediators of gene-environment interactions and central players in the intricate processes of brain development and function (Lossi et al., 2024). Among them, DNA methylation patterns play a crucial role in the proliferation and differentiation of neural stem cells, helping to establish and maintain neuronal identity while contributing to the diversity of neuronal subtypes in the brain (Sun et al., 2021; Lister et al., 2013). DNA methylation has been implicated in several neurodevelopmental disorders, such as autism spectrum disorder (ASD) (Jiang et al., 2022), attention deficit hyperactivity disorder (ADHD) (Carvalho et al., 2023), and Tourette syndrome (TS) (Pagliaroli et al., 2016). However, no studies to date have investigated DNA methylation in DCD. Some research suggests that environmental exposures during fetal life and early childhood, such as air pollution, can induce long-lasting changes in DNA methylation patterns, thereby affecting neurodevelopmental trajectories (Lossi et al., 2024; Broséus et al., 2024). For example, prenatal exposure to PM_10_ has been associated with differential methylation of genes involved in neurodevelopment, which may subsequently impact cognitive and motor function in offspring (Feil et al., 2023). Therefore, further exploration of the epigenetic alterations in DCD, particularly the role of DNA methylation, could provide critical insights into its underlying molecular mechanisms and new perspectives for future precise intervention and therapeutic strategies.

Given the critical role of epigenetic modifications in neurodevelopment, this study aimed to investigate the DNA methylation patterns associated with DCD in children to identify potential epigenetic biomarkers related to motor development. We first performed genome-wide DNA methylation analysis to screen for differentially methylated probes associated with DCD. These probes were then validated in a larger population using MethylTarget™ sequencing, and their associations with motor development scores were assessed. Our findings provide new insights into the epigenetic mechanisms underlying DCD and suggest potential biomarkers for early diagnosis.

## Methods

### Study population

Children who visited Shanghai First Maternity and Infant Hospital with suspected motor coordination disorder were assessed using the Movement Assessment Battery for Children-Second Edition (MABC-2). In combination with the DSM-5 (Diagnostic and statistical manual, 2013) diagnostic criteria, the participants were assigned to DCD group according to the diagnostic results. Age-matched healthy children were selected as controls. Peripheral blood samples were collected from all enrolled children for genome-wide DNA methylation analysis. The other 41 participants were from a previous cohort study conducted at the Shanghai First Maternity and Infant Hospital. Motor development at 1 year of age in 41 participants was assessed using Bayley Scales of Infant and Toddler Development, Third Edition (BSID-III), and peripheral blood was collected for MethylTarget™ sequencing. The study was approved by the Ethic Committee of Shanghai First Maternity and Infant Hospital (KS1630). All information acquired was kept confidential and was only accessible by the researchers.

### DCD diagnosis

In this study, the diagnosis of DCD was based on both the MABC-2 scores and the criteria outlined in DSM-5. According to the DSM-5 (Diagnostic and Statistical Manual, 2013), DCD should be diagnosed based on the following criteria: 1) acquisition and execution of coordinated motor skills are below the expected level for age, given the opportunity for skill learning; 2) motor skill difficulties significantly interfere with activities of daily living and impact academic/school productivity, prevocational and vocational activities, leisure, and play; 3) onset is in the early developmental period; and 4) motor skill difficulties are not better explained by intellectual or visual impairment or other neurological conditions that affect movement. Additionally, children with co-occurring ASD, ADHD, or learning disorders (LD) were excluded from the study.

### Neurodevelopment measurements

The MABC-2 is a widely used diagnostic tool for assessing DCD (Henderson et al., 2007). The test consists of eight items and the recorded raw data are standardized and converted into a standard score of 1–19, reflecting three motor subtests (manual dexterity, aiming and catching, and balance), which represent fine motor skills, gross motor skills, and balance abilities, respectively. Finally, the standardized scores from each subscale are summed to calculate the total MABC-2 score.

The BSID-III is a widely utilized assessment tool for evaluating neurodevelopment in children up to 42 months of age. In this study, we included scores from motor (gross and fine motor) domain. The motor subscale may be useful for describing and assessing motor function, especially for general developmental assessment to identify early motor dysfunction. Frijters et al. (Frijters et al., 2010) have demonstrated that it had a good correlation with MABC-2 results in children aged 36–48 months. As described previously (Hua et al., 2019), the BSID-III was used to assess motor development at 1 year of age in 41 participants. These assessments were conducted by trained professionals. The motor domain composite scores were standardized according to the standard usage of the tool.

### Infinium human methylation 850K BeadChip

Genome-wide DNA methylation analysis was conducted using the Infinium Human Methylation 850K BeadChip (Illumina). Genomic DNA was bisulfite-treated using the EZ DNA Methylation Kit (Zymo Research) according to the manufacturer’s protocol. The treated DNA samples were then hybridized to the BeadChip following the Illumina Infinium HD Methylation Protocol. Raw intensity data (IDAT files) generated from the BeadChip were processed using the ChAMP (Tian et al., 2017) package (version 2.14.0) in R, with the human genome build 19 (hg19) as a reference genome for annotation. DNA methylation levels were represented as β values, ranging from 0 (completely unmethylated) to 1 (fully methylated) for each CpG site. Probes with a detection *P* value >0.01 and located on the X and Y chromosomes were excluded. Additionally, SNP-related probes and multi-hit probes were removed. To correct for Infinium type I and type II probe bias, the BMIQ (Beta MIxture Quantile dilation) algorithm was applied. The final data set for analysis comprised methylation data from 723838 probes. To assess the presence of variation and potential batch effects in the methylation data, we performed singular value decomposition (SVD) analysis and generated a scree plot to visualize the proportion of variance explained by each principal component, thereby identifying potential sources of variation. Additionally, a quantile-quantile (QQ) plot of observed versus expected–log10(*p*) values was generated to evaluate the inflation of test statistics and detect any systematic bias. To control for cell type heterogeneity in blood samples, we applied a reference-based cell composition correction using the champ.refbase.fix function in the ChAMP package in R with default parameters, which estimates and adjusts for the proportions of major blood cell types (CD8^+^ T cells, CD4^+^ T cells, natural killer cells, B cells, monocytes, and granulocytes) based on the method by Houseman et al. (Houseman et al., 2012). The estimated proportions of these cell types for each group are summarized in Supplementary Table S1.

### MethylTarget™ sequencing

MethylTarget™ sequencing (Genesky Biotechnologies Inc., Shanghai, China) was used to assess CpG site methylation levels. Following quality control of genomic DNA, target probes primers were designed and single-site PCR conditions were optimized. The primer sequences for target probes are shown in Supplementary Table S2. Optimized primers were combined into a multiplex PCR panel. After bisulfite conversion, the multiplex PCR panel was used for amplification, ensuring balanced target site products. Indexed primers were then used for PCR to introduce Illumina-compatible tags and sequencing libraries were constructed. Finally, after fragment size verification using Agilent 2100 bioanalyzer, sequencing was performed on the Illumina Hiseq platform (2 × 150 bp) to generate FastQ data.

### Statistical analysis

Statistical analysis was performed using R software (version 4.4.3) and SPSS 26.0 (IBM, Armonk, NY, United States). DMPs between groups were identified using the champ.DMP function in the ChAMP package (version 2.14.0). Given the relatively limited sample size, we applied selection criteria based on both unadjusted statistical significance and effect size. Specifically, DMPs were defined as CpG sites with an unadjusted *P* value <0.05 and an absolute methylation difference (Δβ) > 0.1. This approach has been successfully applied in previous studies to identify DMPs in small samples (Maltby et al., 2020; Imran et al., 2021; Yao et al., 2021; Maltby et al., 2017). DMRs were identified using the champ.DMR function implemented in the ChAMP R package. We applied two algorithms within this function: Bumphunter and ProbeLasso. For Bumphunter, the minimum number of CpGs per DMR was set to ≥2, and the maximum DMR length was restricted to less than 300 bp. For ProbeLasso, default settings were used as provided by the ChAMP package. The DMRs detected by these two algorithms were compared to evaluate the consistency and robustness of the results across different computational methods.

To assess the genomic distribution of DMPs, we compared the numbers of total, hypermethylated, and hypomethylated DMPs in gene-related and CpG island-related regions with the background distribution of all probes on the Illumina 850K array using Fisher’s exact test. Multiple testing correction was performed using the Benjamini–Hochberg (BH) method and an adjusted *P* value <0.05 was considered statistically significant. For the chromosomal distribution analysis, a chi-squared goodness-of-fit test was used to compare the observed number of DMPs per chromosome with the expected distribution based on the total number of probes per chromosome on the 850K array.

Functional enrichment analysis, including Gene Ontology (GO) (Ashburner et al., 2000) analysis and Kyoto Encyclopedia of Genes and Genomes (KEGG) (Kanehisa et al., 2008) pathway analysis, was performed using the Over-Representation Analysis (ORA) method implemented in the R package clusterProfiler (version 3.12.0) (Yu et al., 2012) and the results were visualized using the SRplot platform (www.bioinformatics.com.cn).

In the replication phase, the associations between methylation levels at candidate DMPs and motor outcomes (total motor, gross motor, and fine motor scores) were evaluated using univariate and multiple linear regression models. For the multiple regression models, covariates included maternal age at delivery, child’s gender, and birth weight. Maternal age was grouped into two categories: “<35” and “≥35” years. Effect estimates were presented as standardized β coefficients with 95% confidence intervals (CI). A two-tailed *P* value <0.05 was considered statistically significant. Sensitivity analysis was conducted by adjusting for the covariates to assess the robustness of the findings.

## Results

### Characteristics of the participants

The diagnosis of DCD was performed according to DSM-5 criteria, and 9 of the tested children had confirmed DCD. One child over 7 years of age was excluded based on age (3–7 years) and the remaining participants were then matched in groups based on age and gender. Of the 16 participants, 8 children with DCD and 8 children without DCD had mean ages of 3.25 ± 0.71 and 3.75 ± 1.39 years, respectively, with no statistically significant difference between the groups (t = 0.907, *P* = 0.3795). The sex distribution was identical between the DCD and control groups, with each group including 5 boys and 3 girls. The MABC-2 score percentiles were significantly lower in the DCD group (6.30 ± 8.72) compared to the control group (74.38 ± 19.19), with a statistically significant difference (t = 9.134, *P* < 0.0001). Peripheral blood was collected from enrolled children for methylation data analysis (Table 1).

**TABLE 1:** Characteristics of the participants.

Characteristics	Study cohort for genome-wide DNA methylation analysis (n = 16)		*P* value^a^
	Control	DCD	
Participants	8	8	-
MABC-2 score percentile [mean (SD)]	74.38 (19.19)	6.30 (8.72)	<0.0001
Age (years) [mean (SD)]	3.75 (1.39)	3.25 (0.71)	0.3795
Gender [n (%)]			
Male	5 (62.5%)	5 (62.5%)	-
Female	3 (37.5%)	3 (37.5%)	-

Abbreviation: SD, standard deviation.

^a^ The *P* value is calculated from two sample t-test.

### DMPs in children with DCD

Genome-wide DNA methylation profiling of 16 children was detected using 850K BeadChip. To assess potential technical batch effects, SVD analysis was conducted using variables including slide, array, plate, well, start_date, and sample_group. As shown in Supplementary Figure S1A, only the sample_group variable was significantly associated with the first principal component (PC1, *P* < 0.05), whereas all technical variables were not significantly associated (*P* > 0.05). A scree plot (Supplementary Figure S1B) further demonstrated that the first three principal components explained 41.82% of the total variance. These findings suggested that the primary source of variation was biological rather than technical. Additionally, the QQ plot has been presented in Supplementary Figure S1C. The observed genomic inflation factor (λ = 1.168) falls within acceptable limits, indicating no substantial inflation or systematic technical bias in the test statistics.

**FIGURE 1:**
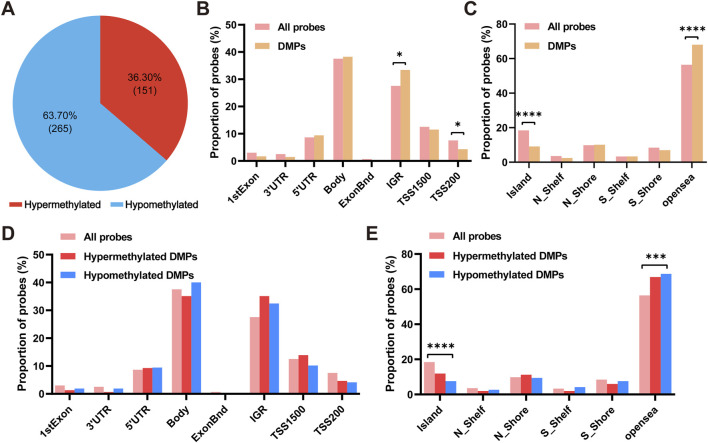
Characteristics of DMPs in DCD. **(A)** Proportion of hypermethylated (red) and hypomethylated (blue) DMPs. **(B,C)** The category of genomic locations for all probes and DMPs. **(D,E)**The category of genomic locations for hypermethylated (red) or hypomethylated (blue) DMPs. 1stExon, first exonic region on the gene; 3′UTR, between the stop codon and poly A signal; 5′UTR, within the 5′untranslated region and between the TSS and the ATG start site; body, gene region; IGR, intergenic region; TSS1500, 200–1500 bases upstream of the transcriptional start site (TSS); TSS200, 0–200 bases upstream of the TSS. Island, CpG island; Shore, 0–2 kb from CpG island; Shelf, 2–4 kb from CpG island; opensea, other genomic regions. Fisher’s exact test, *, *P* < 0.05; ***, *P* < 0.001; ****, *P* < 0.0001.

**FIGURE 2:**
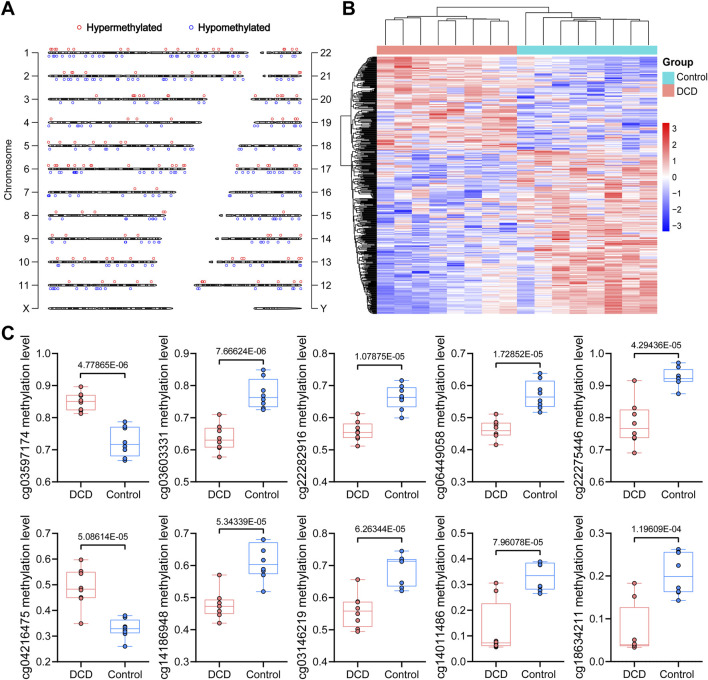
Chromosomal distribution and methylation patterns of DMPs in DCD. **(A)** Distribution of DMPs across Chromosomes. **(B)** Heatmap of hierarchical clustering analysis for samples according to DMPs. **(C)** Top 10 DMPs ranked by *P*-value.

Based on these results, we subsequently performed differential methylation analysis between groups. As shown in Figure 1A, a total of 416 DMPs, including 151 hypermethylated DMPs (36.30%) and 265 hypomethylated DMPs (63.70%), were identified in the DCD group compared with the control group. 277 of these DMPs had UCSC RefGene Name, mapping to 238 unique genes, while other DMPs were not annotated to specific genes. The RefGene name and the location of each DMP in a gene or chromosome are provided in Supplementary Table S3 and Supplementary Figure S2. We next examined the genomic distribution of these DMPs. As shown in Figure 1B, DMPs were most frequently located in gene bodies (Body, 38.22%) and intergenic regions (IGR, 33.41%). However, Fisher’s exact test indicated that DMPs were significantly enriched in IGR (adjusted *P* = 0.047) but not in Body regions. In contrast, DMPs were significantly depleted in TSS200 regions (adjusted *P* = 0.047) (Figure 1B; Supplementary Table S4). Similarly, within CpG island-related regions, DMPs were significantly enriched in opensea regions (adjusted *P* = 4.36 × 10^−6^) and depleted in CpG islands (adjusted *P* = 1.04 × 10^−6^) (Figure 1C; Supplementary Table S4). Further stratified analysis showed that hypomethylated DMPs were also enriched in opensea regions (adjusted *P* = 1.56 × 10^−4^) and depleted in CpG islands (adjusted *P* = 4.12 × 10^−6^), while no significant enrichment or depletion was observed for hypermethylated DMPs in any genomic or CpG island-related region (Figures 1D,E; Supplementary Tables S5, S6). The distribution of DMPs varied among chromosomes, with the highest number observed on chromosome 1, chromosome 6, and chromosome 2 (Figure 2A; Supplementary Table S7). However, a chi-squared goodness-of-fit test showed that this distribution did not significantly differ from that of all detected probes (χ^2^ = 26.13, df = 21, *P* = 0.2016), indicating no significant chromosomal enrichment. Additionally, a heatmap showing the methylation levels of all DMPs is presented in Figure 2B. The top 10 DMPs ranked by *P* value are shown in Figure 2C, and those ranked by absolute methylation difference are shown in Supplementary Figure S3.

### DMRs in children with DCD

**FIGURE 3:**
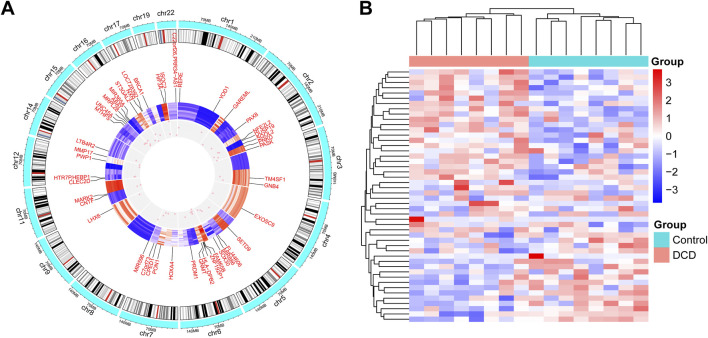
Characteristics of DMRs identified by the Bumphunter algorithm in DCD. **(A)** Circos plot of DMRs. **(B)** Heatmap of hierarchical clustering analysis for samples according to DMRs.

A total of 48 DMRs were identified between the DCD and control groups using the Bumphunter algorithm, mapping to 48 unique genes (Supplementary Table S8). The distribution and methylation level of these DMRs were visualized by a circos plot and heatmap (Figures 3A,B).Among these, 12 genes overlapped with those annotated by DMPs. A hypergeometric test showed that the overlap is significantly more than expected by chance (*P* = 2.91 × 10^−13^). To assess the robustness of DMR detection, we further applied the ProbeLasso algorithm implemented in the ChAMP package with default parameters. This analysis identified 22 DMRs mapping to 24 genes (Supplementary Figure S4A; Supplementary Table S9). Among these, three genes (ABAT, NXN, and MIR365A) were consistently identified by both ProbeLasso and Bumphunter (Supplementary Figure S4B), suggesting that these genes represent robust DMR signals across different computational strategies. This cross-validation supports the reliability of the observed regional methylation changes. For subsequent analyses, we used the DMRs identified by the Bumphunter algorithm.

### GO and KEGG pathway enrichment analysis

**FIGURE 4:**
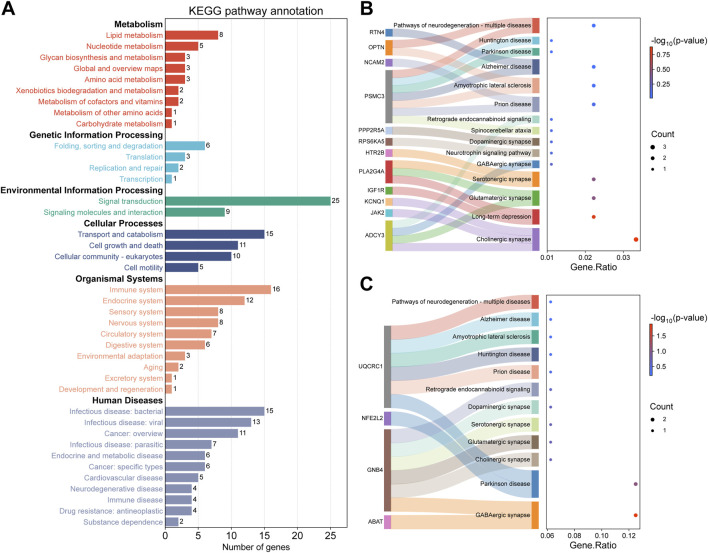
KEGG pathway enrichment analysis. **(A)** KEGG pathway classification of DMPs-associated genes. **(B)** KEGG pathways related to the nervous system and neurodegenerative diseases enriched in DMPs-associated genes. **(C)** KEGG pathways related to the nervous system and neurodegenerative diseases enriched in DMRs-associated genes.

To explore the functional annotation of DMPs and DMRs, we performed GO term and KEGG pathway enrichment analyses on genes associated with DMPs and DMRs using the ORA method implemented in the R package clusterProfiler. The DMPs-associated genes were enriched in 3385 GO terms and 204 KEGG pathways, while the DMRs-associated genes were enriched in 1356 GO terms and 71 KEGG pathways. To further classify the enriched pathways, we performed KEGG pathway classification analysis (Figure 4A; Supplementary Figure S5C). We found that DMPs-associated genes were enriched in 15 KEGG pathways associated with the nervous system and neurodegenerative diseases (Figure 4B), whereas DMRs-associated genes were enriched in 12 such pathways (Figure 4C). Additionally, the top five enriched GO terms in each category (biological process, cellular component, and molecular function) for DMPs- and DMRs-associated genes are presented in Supplementary Figures S5A, S5B.

### Screening of key DMPs in DCD

To investigate the association between DNA methylation and motor performance in children, we analyzed the correlation between the methylation levels of DMPs and MABC-2 scores. A total of 251 DMPs were significantly associated with motor performance. For further validation, we prioritized DMPs based on their statistical significance, functional relevance, and involvement in biological pathways. We identified 42 key DMPs mapped to genes involved in neurodevelopment and motor function (Table 2). For example, *LIN28* and *ATXN7* have been implicated in neurogenesis and brain development (Wang and Li, 2024; Niewiadomska-Cimicka and Trottier, 2019). *IGF1R* plays a crucial role in synaptic plasticity and complex cognitive functions (Cardoso et al., 2021). *NCK1* is essential for neuronal connectivity and signaling (Fawcett et al., 2007) and *TNFAIP3* is involved in regulating microglia activation and neuroinflammation (Voet et al., 2018). Additionally, the *COL5A1* gene encodes the α1 chain of type V collagen, which is crucial for musculoskeletal development (Chandrasekaran et al., 2024).

**TABLE 2:** Genomic characteristics of 42 Key DMPs in DCD.

Probe	Chr	MAPINFO	UCSC RefGene Name	UCSC RefGene Group	UCSC RefGene Name Annovar	Type
cg03597174	17	27359875		IGR	SEZ6, PIPOX	Hypermethylated
cg03603331	8	134104425	TG	Body	SLA,TG	Hypomethylated
cg22282916	22	43357102	PACSIN2	TSS1500	PACSIN2	Hypomethylated
cg06449058	6	158991790	TMEM181	Body	TMEM181	Hypomethylated
cg22275446	3	63883664	ATXN7	TSS1500	ATXN7	Hypomethylated
cg04216475	6	159520036		IGR	TAGAP, LOC101929122	Hypermethylated
cg14186948	9	137723167	COL5A1	Body	LOC101448202	Hypomethylated
cg03146219	11	71189514	NADSYN1	Body	NADSYN1	Hypomethylated
cg18634211	1	26737262	LIN28	TSS200	LIN28A	Hypomethylated
cg23875752	11	71189385	NADSYN1	Body	NADSYN1	Hypomethylated
cg22110428	19	51980908	CEACAM18	TSS1500	CEACAM18	Hypomethylated
cg11883129	2	48569029	FOXN2	5′UTR	FOXN2	Hypomethylated
cg12126686	19	35821634	CD22	5′UTR	CD22	Hypermethylated
cg11024728	7	1425807		IGR	UNCX, MICALL2	Hypomethylated
cg25937052	4	41649731	LIMCH1	Body	LIMCH1	Hypomethylated
cg04959182	2	86770055	CHMP3	Body	CHMP3, RNF103-CHMP3	Hypomethylated
cg08314849	2	25192865	DNAJC27	Body	DNAJC27	Hypomethylated
cg24419094	2	10266986	RRM2	Body	RRM2	Hypomethylated
cg10193422	14	65537522	MAX	Body	MAX	Hypermethylated
cg09238666	16	66584358	TK2	TSS200	TK2	Hypomethylated
cg18187326	10	120873428	FAM45A	Body	FAM45BP	Hypomethylated
cg11543899	9	20607066	MLLT3	Body	MLLT3	Hypomethylated
cg10283362	21	37501846		IGR	LOC100133286, CBR3-AS1	Hypomethylated
cg14933993	21	45341553	AGPAT3	5′UTR	AGPAT3	Hypermethylated
cg17073989	1	90321453	LRRC8D	5′UTR	LRRC8D	Hypermethylated
cg11053414	1	85135713	SSX2IP	Body	SSX2IP	Hypomethylated
cg20049730	2	147075656		IGR	TEX41, PABPC1P2	Hypomethylated
cg02152351	6	8436296	SLC35B3	TSS1500	LOC100506207	Hypermethylated
cg16113883	6	138190021	TNFAIP3	5′UTR	TNFAIP3	Hypermethylated
cg11112615	6	2970624	SERPINB6	5′UTR	SERPINB6	Hypomethylated
cg13388253	19	51505507	KLK8	TSS1500	KLK8, KLK9	Hypermethylated
cg06571226	1	230439016		IGR	GALNT2, PGBD5	Hypomethylated
cg12709880	18	21163172	NPC1	Body	NPC1	Hypomethylated
cg12437013	13	114161939	TMCO3	Body	TMCO3	Hypomethylated
cg13860573	3	136649005	NCK1	TSS1500	NCK1	Hypomethylated
cg16092154	1	55414179		IGR	DHCR24, TMEM61	Hypomethylated
cg01684255	3	194142667	ATP13A3	Body	ATP13A3	Hypomethylated
cg11968956	6	119441387	FAM184A	5′UTR	FAM184A	Hypomethylated
cg16667827	17	57803298	VMP1	5′UTR	VMP1	Hypomethylated
cg08920032	15	99,332,004	IGF1R	Body	IGF1R	Hypomethylated
cg05986449	2	157320849	GPD2	5′UTR	GPD2	Hypomethylated
cg22082780	1	68452167		IGR	GNG12-AS1	Hypomethylated

### Population-based replication

**FIGURE 5:**
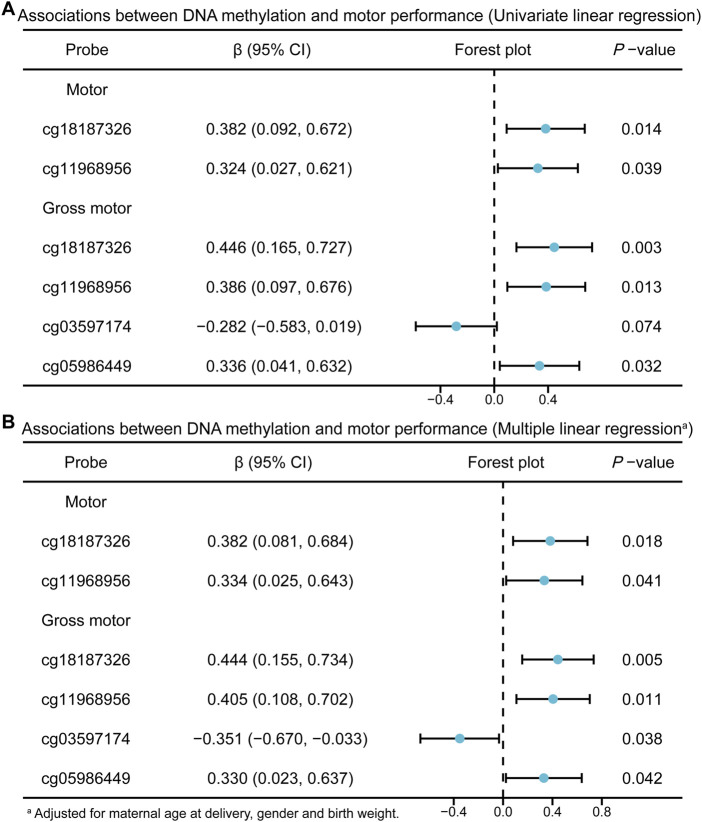
Associations between DNA methylation and motor performance in replication phase. **(A)** Univariate linear regression. **(B)** Multiple linear regression.

Next, we validated the associations between the methylation levels of key DMPs and motor performance in a larger population using MethylTarget™ sequencing. Table 3 shows the characteristics of the participants in the replication phase. As shown in Figure 5 and Supplementary Material 2, after adjusting for potential confounders, methylation levels at cg18187326 (*FAM45A*, chr10:120873428) and cg11968956 (*FAM184A*, chr6:119441387) remained significantly associated with total motor performance (cg18187326, adjusted β = 0.382, *P* = 0.018; cg11968956, adjusted β = 0.334, *P* = 0.041). For gross motor, higher methylation levels at cg18187326 and cg11968956 were also positively correlated with better gross motor performance (cg18187326, adjusted β = 0.444, *P* = 0.005; cg11968956, adjusted β = 0.405, *P* = 0.011). In addition, cg05986449 (*GPD2*, chr2:157320849) was significantly associated with improved gross motor performance (adjusted β = 0.330, *P* = 0.042). Notably, cg03597174 (*SEZ6* (dist = 26417), *PIPOX* (dist = 10043), chr17:27359875) showed a negative association with gross motor performance, suggesting that increased methylation level at cg03597174 may be linked to poorer gross motor performance (adjusted β = −0.351, *P* = 0.038). However, no significant associations were observed between methylation levels at these DMPs and fine motor performance. The genomic locations and other annotations of these four DMPs are summarized in Table 4.

**TABLE 3:** Basic characteristics of the population in replication phase.

Characteristics	*n*	Mean ± SD or (%)
Participants	41	
Birth weight (g)^a^	41	3405.37 ± 453.93
Gender^b^		
Boys	19	46.34
Girls	22	53.66
Mode of delivery^b^		
Cesarean delivery	41	100.00
Maternal age at delivery (years)^b^		
**≥ 35**	18	43.90
**< 35**	23	56.10

Abbreviations: SD, standard deviation.

^a^ Data were presented as mean ± SD, for continuous variables.

^b^ Number and percentage/proportion for categorical variables.

Bold values in “Maternal age at delivery” were unintentional. Maternal age was categorized as <35 and ≥35 years, as described in the Methods.

**TABLE 4:** Location of the selected DMPs in population-based replication phase.

Probe	Chr	MAPINFO	UCSC RefGene Name	UCSC RefGeneGroup	UCSC RefGene Name Annovar	UCSC RefGene Group Annovar	UCSC CpG Island	Type
cg18187326	10	120873428	FAM45A	Body	FAM45BP	ncRNA intronic	opensea	Hypomethylated
cg11968956	6	119441387	FAM184A	5′UTR	FAM184A	intronic	opensea	Hypomethylated
cg05986449	2	157320849	GPD2	5′UTR	GPD2	intronic	opensea	Hypomethylated
cg03597174	17	27359875		IGR	SEZ6 (dist = 26417), PIPOX (dist = 10043)	intergenic	opensea	Hypermethylated

### Sensitivity analysis

We conducted sensitivity analysis to examine the robustness of the associations between methylation levels at cg18187326, cg11968956, cg03597174, and cg05986449 and motor performance. Different combinations of covariates, including maternal age at delivery, gender, and birth weight, were tested. The results remained consistent with the main analysis, supporting the stability of the observed associations (Table 5).

**TABLE 5:** Association between DNA methylation and motor performance in different adjusted models using multiple linear regression.

Probe	Adjusted^a^		Adjusted^b^		Adjusted^c^	
	β (95% *CI*)	*P-*value	β (95% *CI*)	*P-*value	β (95% *CI*)	*P-*value
Motor						
cg18187326	0.384 (0.087, 0.682)	0.016	0.381 (0.084, 0.677)	0.016	0.382 (0.085, 0.680)	0.016
cg11968956	0.327 (0.022, 0.632)	0.042	0.334 (0.029, 0.638)	0.038	0.334 (0.030, 0.639)	0.038
Gross motor						
cg18187326	0.450 (0.162, 0.738)	0.004	0.444 (0.159, 0.729)	0.004	0.444 (0.159, 0.730)	0.004
cg11968956	0.392 (0.095, 0.689)	0.014	0.405 (0.113, 0.698)	0.010	0.405 (0.112, 0.698)	0.010
cg03597174	−0.282 (−0.593, 0.029)	0.084	−0.329 (−0.638, −0.021)	0.043	−0.348 (−0.662, −0.034)	0.036
cg05986449	0.338 (0.033, 0.643)	0.036	0.331 (0.028, 0.634)	0.039	0.329 (0.028, 0.630)	0.039

^a^ Adjustment: maternal age at delivery and gender.

^b^ Adjustment: maternal age at delivery and birth weight.

^c^ Adjustment: gender and birth weight.

## Discussion

DCD is a common but under-recognized neurodevelopmental disorder characterized by impaired motor coordination, which often persists into adolescence and adulthood, leading to a range of adverse psychosocial consequences (Biotteau et al., 2020; Blank et al., 2019). However, the underlying biological mechanisms of DCD remain largely unclear. Growing evidence suggests that epigenetic regulation, particularly DNA methylation, plays an important role in brain development and neurobehavioral regulation (Levy et al., 2022; Godler and Amor, 2019; Ehlinger et al., 2023). Therefore, exploring the DNA methylation profile of individuals with DCD may provide novel insights into its molecular etiology and identify potential biomarkers for early diagnosis and intervention. In the present study, we conducted genome-wide DNA methylation analysis using peripheral blood samples from children with and without DCD. A total of 416 DMPs and 48 DMRs were identified, of which 42 key DMPs were selected for further replication in a larger population. Notably, methylation levels at cg18187326 and cg11968956 were significantly associated with both total and gross motor scores. Additionally, cg03597174 was negatively associated with gross motor performance, whereas cg05986449 showed a positive correlation. This study is the first to investigate DNA methylation patterns in children with DCD, providing novel epigenetic insights into its potential molecular mechanisms.

The CpG site cg18187326 is annotated as *FAM45A* (Family with sequence similarity 45 member A) gene. According to UCSC RefGene Group annotation, it lies within the gene body, while Annovar annotates it as located in a ncRNA intronic region. *FAM45A*, also known as *DENND10*, encodes a protein belonging to the DENND protein family of guanine nucleotide exchange factors targeting Rabs. It is involved in late endosome homeostasis and exosome biogenesis (Zhang et al., 2019). As a part of the Commander complex, FAM45A dysfunction may indirectly affect endocellular trafficking processes associated with Ritscher-Schinzel, a multisystem developmental disorder characterized by abnormal craniofacial features as well as cerebellar hypoplasia, thus playing a role in the development of the disease (Laulumaa et al., 2024; Healy et al., 2023). The CpG site cg11968956 is located within the 5′UTR of the *FAM184A* (Family with sequence similarity 184 member A) gene based on UCSC RefGene Group annotation and within an intronic region according to Annovar. *FAM184A* is highly expressed in the human cerebral cortex and cerebellum (Uhlén et al., 2015). Previous research had shown that *FAM184A* expression was significantly upregulated following umbilical cord occlusion in a preterm brain injury model, with expression levels positively correlated with the severity of brain injury (Ek et al., 2024), suggesting a potential role in the pathophysiological processes of neural damage. DNA hypomethylation in regulatory regions such as the 5′UTR and introns is often associated with increased gene expression (Anastasiadi et al., 2018). In this study, cg11968956 was hypomethylated in children with DCD. Therefore, this finding suggests that *FAM184A* expression may be elevated in children with DCD, further supporting its potential involvement in the molecular mechanisms underlying neurodevelopmental abnormalities.

In addition, the CpG site cg03597174 is located in an intergenic region near the *SEZ6* (Seizure related 6 homolog) gene and was found to be hypermethylated in children with DCD, showing a negative correlation with gross motor function. The *SEZ6* gene encodes a transmembrane protein specifically localized to neuronal dendrites and plays a critical role in dendritic arborization and synaptogenesis (Osaki et al., 2011). Previous studies have shown that *SEZ6* knockout mice exhibit deficits in motor learning, impaired motor coordination, and spatial memory impairments (Nash et al., 2020; Gunnersen et al., 2007), highlighting its essential role in central nervous system function. Therefore, hypermethylation of cg03597174 may lead to reduced *SEZ6* expression, thereby disrupting the development and integration of neural networks, and consequently contributing to motor function impairments observed in children with DCD. In contrast, cg05986449 was positively associated with gross motor performance and found to be hypomethylated in children with DCD. Cg05986449 is located within the *GPD2* (glycerol-3-phosphate dehydrogenase 2) gene, which is actively expressed in brain tissue and plays a vital role in mitochondrial energy metabolism (Oh et al., 2023) and the regulation of oxidative stress (Ansell et al., 1997). One study reported that regions of the mouse brain with higher synaptic density exhibited elevated *GPD2* activity (Nguyen et al., 2003), suggesting a potential role for *GPD2* in neurotransmission. Moreover, functional impairment of *GPD2* has been associated with neurodevelopmental delay (Daoud et al., 2009).

This study has several limitations. First, the DNA methylation pattern of peripheral blood cannot fully reflect epigenetic changes in brain tissue, although studies have suggested that peripheral methylation markers can serve as surrogate markers of neurological diseases to some extent (Mendonça et al., 2024; Davies et al., 2012). Second, the relatively small sample size of this study may have reduced the statistical power and limited the ability to detect probes reaching significance after multiple testing correction (FDR). Third, although motor development was assessed at 1 year of age using the BSID-III, the replication cohort was not followed up to the age at which DCD can be formally diagnosed. Future studies with larger sample sizes and longer follow-up visits are necessary to verify the predictive value of the identified CpG sites in DCD. In addition, functional experimental studies are needed to elucidate the biological relevance of these epigenetic modifications and their potential causal roles in neurodevelopmental disorders.

## Conclusion

In conclusion, this study identified several key CpG sites associated with DCD, including cg18187326 (*FAM45A*), cg11968956 (*FAM184A*), cg03597174 (*SEZ6*), and cg05986449 (*GPD2*). These epigenetic alterations may influence the expression of genes involved in neurodevelopment, synaptogenesis, and motor function regulation. Our findings provide new insights into the epigenetic mechanisms underlying DCD, suggesting that DNA methylation dysregulation may contribute to its pathogenesis. These findings suggest the potential of DNA methylation markers as biomarkers for early diagnosis and targeted intervention in DCD. Nevertheless, larger cohorts and functional studies are needed to elucidate causal relationships and further elucidate the underlying biological mechanisms.

## Data Availability

The original contributions presented in the study are included in the article/Supplementary Material, further inquiries can be directed to the corresponding authors.
