# Supplementary material for: Epigenetic modifications in developmental coordination disorder: association between DNA methylation and motor performance
Source: Front Cell Dev Biol. 2025 Sep 9;13:1647365. doi: 10.3389/fcell.2025.1647365 (PMC12454365; doi:10.3389/fcell.2025.1647365)
Supplement: Supplementary file 1 [file DataSheet1.docx]

Supplementary Material

## Supplementary Figures


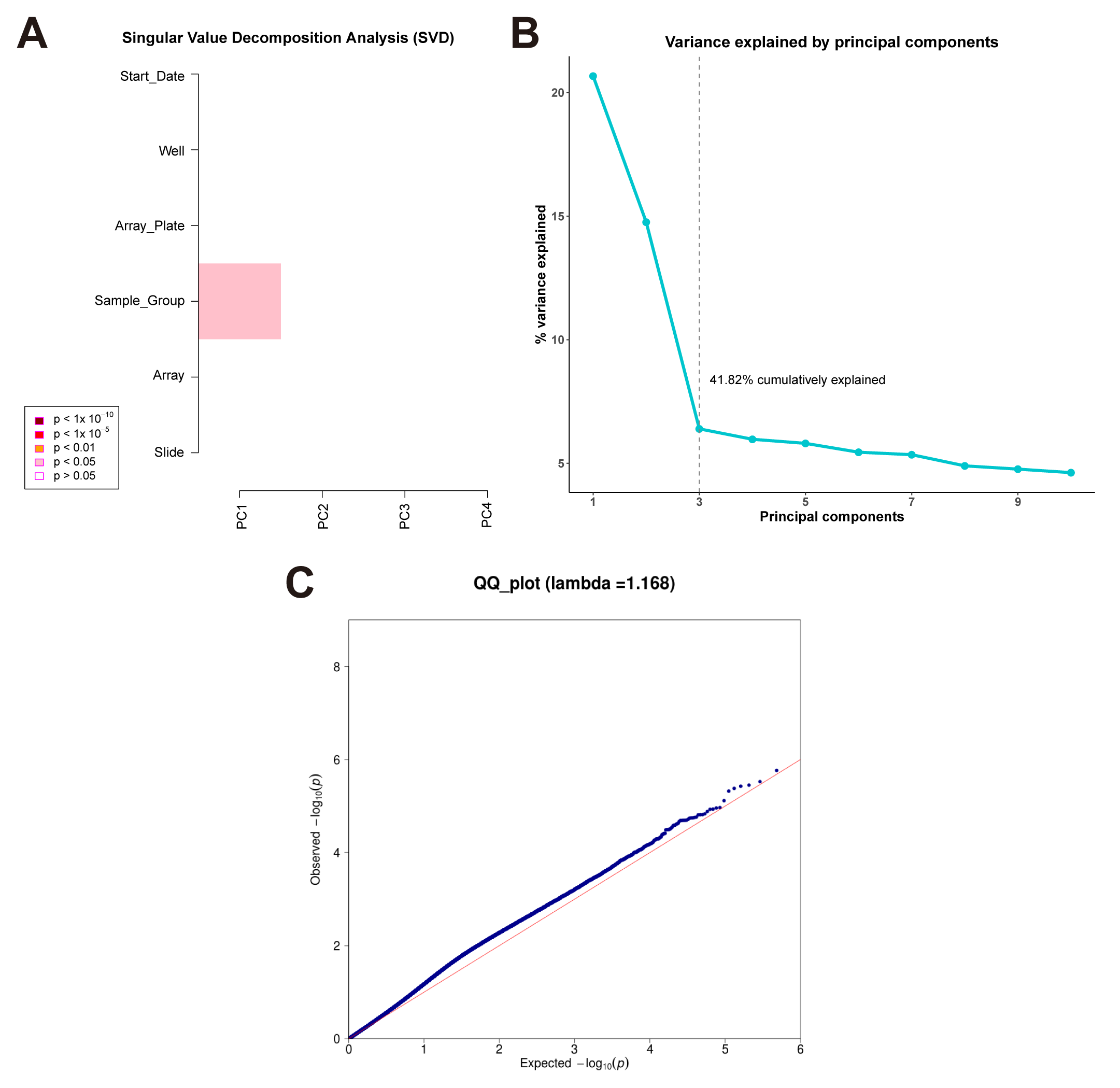


**Supplementary Figure 1. Quality control of DNA methylation data.** (A) Singular value decomposition (SVD) analysis. (B) Scree plot. (C) Quantile–quantile (QQ) plot.


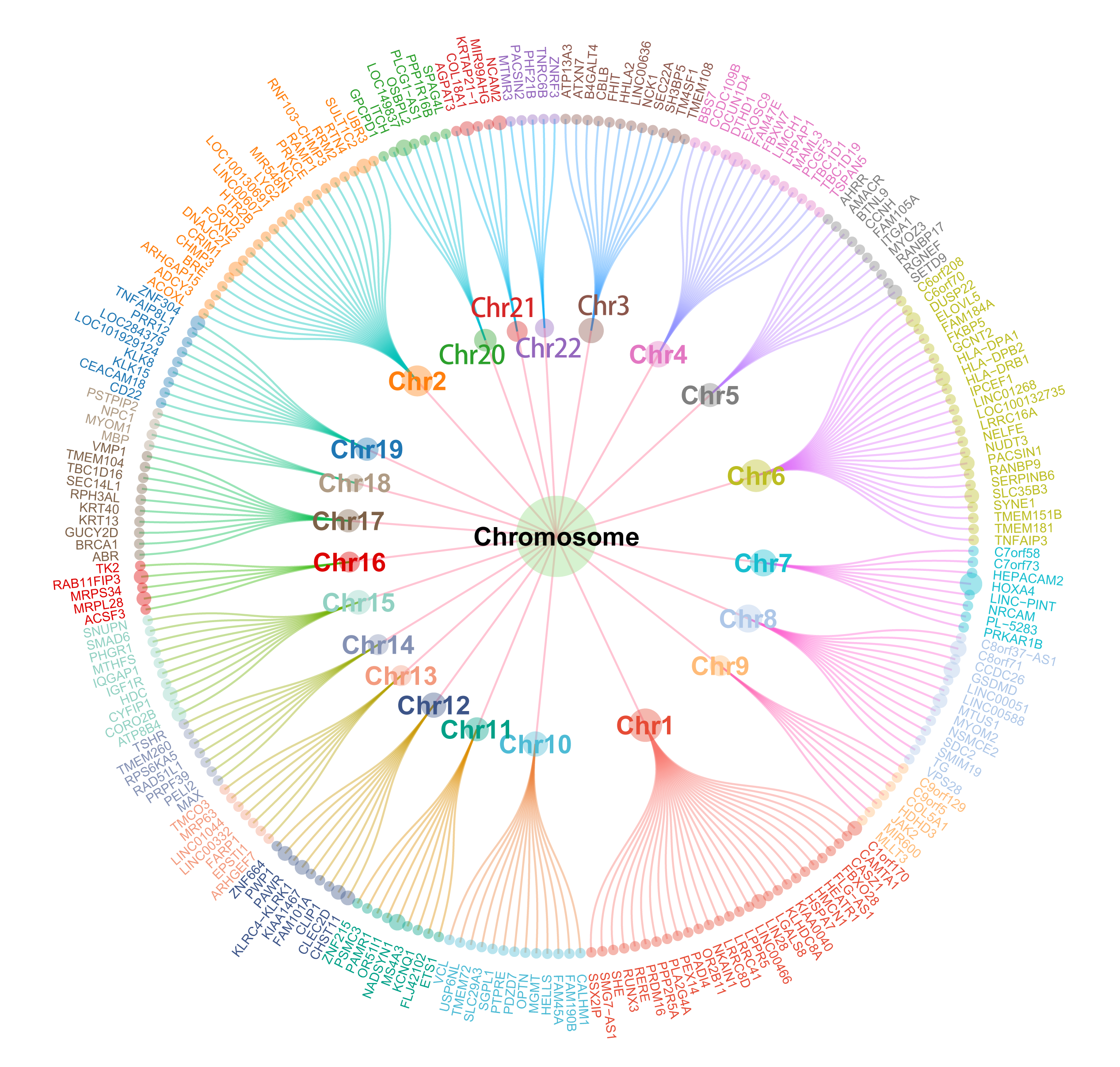


**Supplementary Figure 2.** **Distribution of DMPs in chromosomes and genomic regions.**


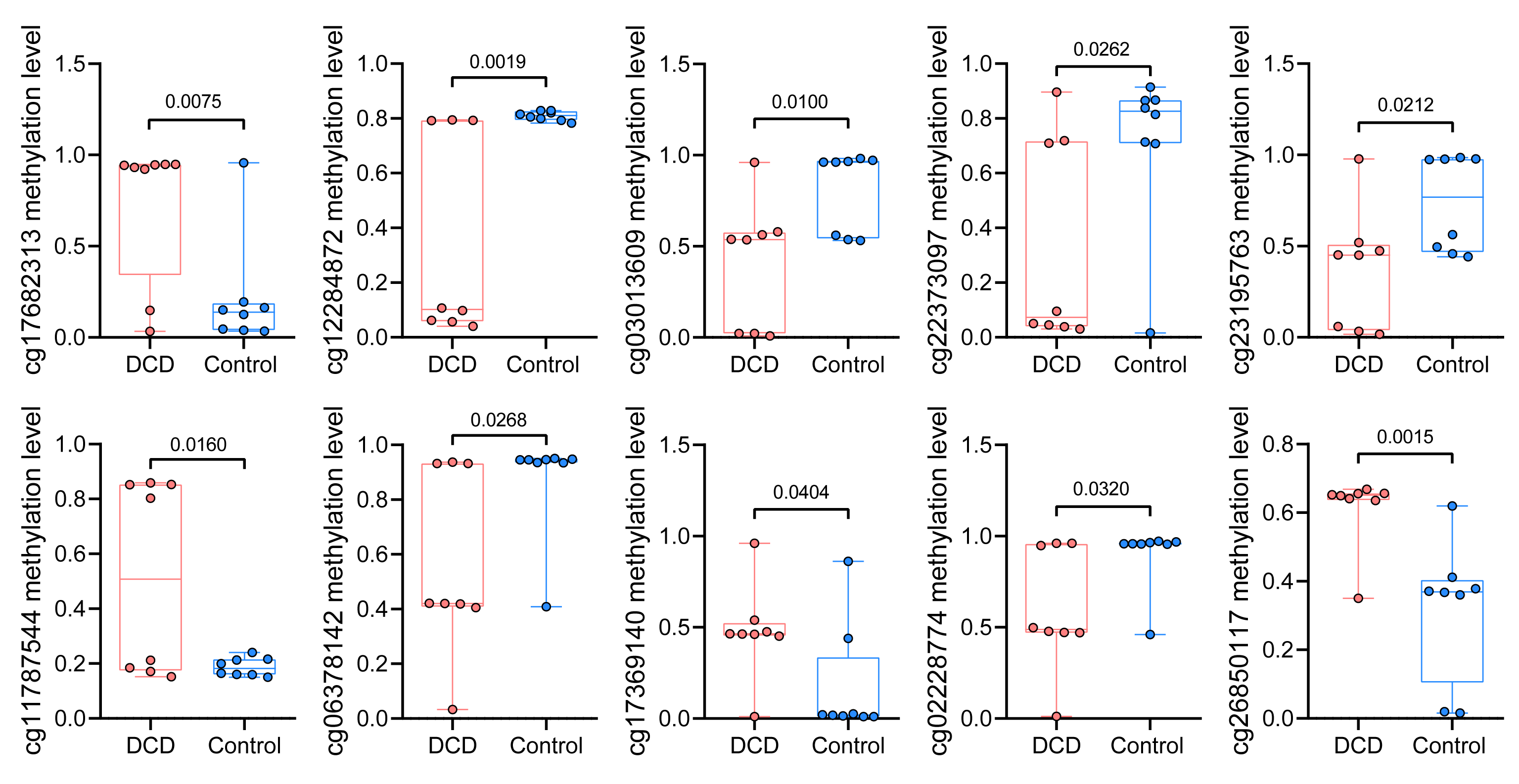


**Supplementary Figure 3. Top 10 DMPs ranked by absolute methylation difference.**


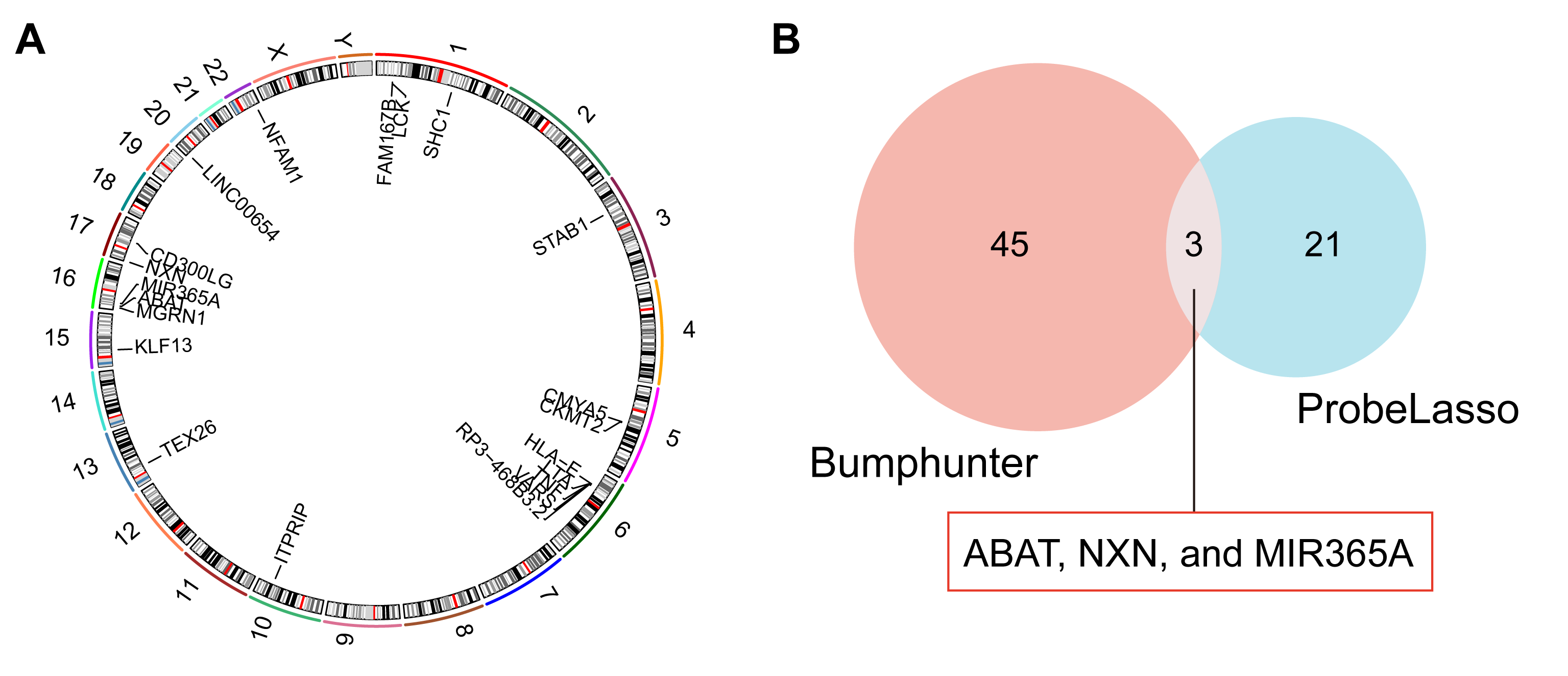


**Supplementary Figure 4.** **Characteristics of DMRs identified by the ProbeLasso algorithm in DCD.** (A) Circos plot of DMRs. (B) Venn diagram showing the overlap of genes annotated by DMRs identified using Bumphunter and ProbeLasso algorithms.


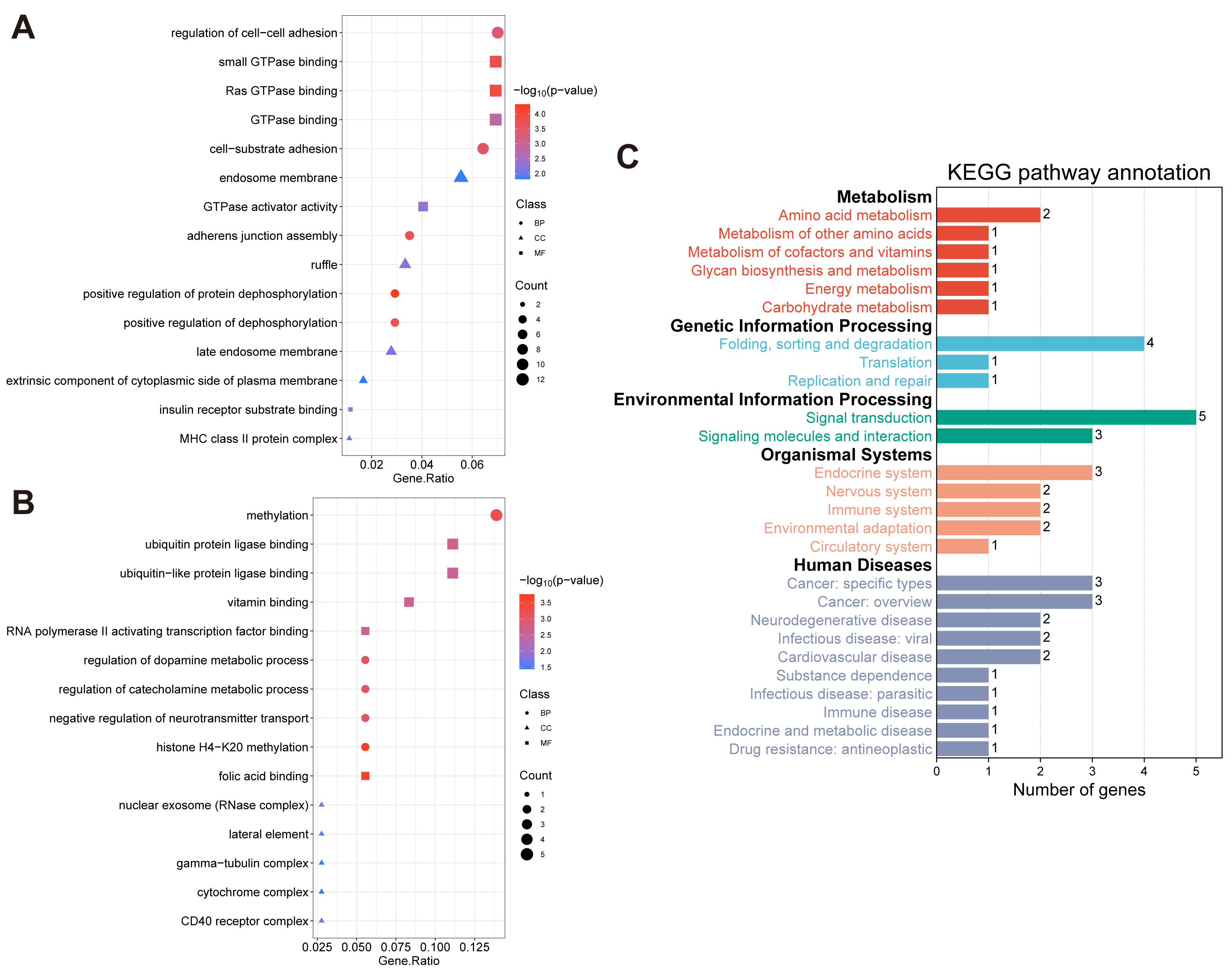


**Supplementary Figure 5. GO and KEGG pathway enrichment analysis.** (A) The top 5 enriched GO terms in each category (biological process, cellular component, and molecular function) of DMPs-associated genes. (B) The top 5 enriched GO terms in each category (biological process, cellular component, and molecular function) of DMRs-associated genes. (C) KEGG pathway classification of DMRs-associated genes.

**Supplementary Tables**

**Supplementary Table 1 Estimated proportions of major blood cell types**

| **Group** | **Sample number** | **CD8+ T cells** | **CD4+ T cells** | **Natural killer cells** | **B cells** | **Monocytes** | **Granulocytes** |
| --- | --- | --- | --- | --- | --- | --- | --- |
| DCD | 0065mabc2 | 0.050249507 | 0.188711983 | 0 | 0.063365567 | 0.052903885 | 0.600517738 |
| DCD | 0067mabc2 | 0.08784936 | 0.234504041 | 1.38778E-17 | 0.159797288 | 0.008782974 | 0.491531845 |
| DCD | 0069mabc2 | 0.072019642 | 0.242184642 | 0 | 0.106496769 | 0.015589521 | 0.557529147 |
| DCD | 0071mabc2 | 0.08183225 | 0.179128559 | 0 | 0.142872635 | 0.02814246 | 0.552877175 |
| DCD | 0073mabc2 | 0.109604716 | 0.180509141 | 0.046130026 | 0.107881205 | 0.030388966 | 0.499962118 |
| DCD | 0077mabc2 | 0.117001105 | 0.099572828 | 0 | 0.096393665 | 0.051967131 | 0.635065271 |
| DCD | 0079mabc2 | 0.096991939 | 0.249993118 | 1.38778E-17 | 0.170203659 | 0.019068134 | 0.440743385 |
| DCD | DCDxp00 | 0.057215018 | 0.207221072 | 0 | 0.089433883 | 0.12095517 | 0.489641054 |
| Control | 0049mabc2 | 0.061470898 | 0.158408481 | 0.047620552 | 0.099380216 | 0.022360931 | 0.568064614 |
| Control | 0053mabc2 | 0.093907274 | 0.297877144 | 0 | 0.115145767 | 0.013959597 | 0.476588862 |
| Control | 0055mabc2 | 0.116592352 | 0.269829396 | 0 | 0.186627804 | 0.024859407 | 0.346981305 |
| Control | 0057mabc2 | 0.147134253 | 0.239527691 | 0 | 0.136098151 | 0.045376257 | 0.418051995 |
| Control | 0075mabc2 | 0.096994723 | 0.266221286 | 0 | 0.153252044 | 0.024447173 | 0.428030852 |
| Control | 0059mabc2 | 0.044681871 | 0.300885876 | 0 | 0.112277381 | 0.0045236 | 0.514459776 |
| Control | 0061mabc2 | 0.196865608 | 0.185986226 | 0.025761258 | 0.121546333 | 0.026434538 | 0.408916267 |
| Control | 0103mabc2 | 0.159302021 | 0.233722895 | 0 | 0.195204563 | 0.014326544 | 0.377514628 |

**Supplementary Table 2 Primer sequences used in MethylTarget sequencing**

| **Probe** | **Forward primer sequence (5’→3’)** | **Reverse primer sequence (5’→3’)** |
| --- | --- | --- |
| cg01684255 | GATTTGYGATTTGGTTTTTGATATGTG | AATTCAATTACTTATCTTCTCTCTCACATAAA |
| cg02152351 | TTTYGGTGAAGATGGGTTTG | ATCCCCAAACCRTCTTAATTTTT |
| cg03146219 | TGGAGGAYGTTYGGAGTTATAGG | ACTTTTCAAACACTACACCCTCAC |
| cg03597174 | GTTTGAGAGGTGAGAGATTGAGAG | AAACCCCTACAAAACTCTCAACA |
| cg03603331 | TTGTGTTGTGTGATTTTTAGTTAGTGGT | TCCACACACACAACCACAAA |
| cg04216475 | TTTATTTAGGTTGGGAGTTGATGTT | AACAACTATACCCTTTACCTTTCCATAC |
| cg04959182 | TTTTGAAGTATTAAATATAGGGTGGATTG | AACTCATAACTCAAATTTTCCTTCAATA |
| cg05986449 | TTTTAAATGGGAATTTTGTTAGTAGTATG | TTTACTCCTCTATACATCTAATAATCCACAAA |
| cg06449058 | GGATTAGATGGTGGGGAAATG | ACCTACACCAACTCCTCTAAACAA |
| cg06571226 | GAATTGTGTTGAGGATTTATTGTTTTT | CAACTATAAATCTATCCACCAAATAATAAAC |
| cg08314849 | GGAATTGAATTTTATTTGTAGGTTGAG | AATTCAATAAAACAAAATTATTACCCACAA |
| cg08920032 | TAGAAATAATTAYGGAGAAATGGTAAAGAAAA | ATAAAACCTATCCTAATCCCTATTCTAACC |
| cg09238666 | TATTYGGAGGATGGATGGTAAA | CTATTACTTCTAACCATTTTAAACAAATTACC |
| cg10193422 | GGGTTTTTGAAGGGGAAGGT | ATACTAACRACTTTACCCTATTCTTCATTC |
| cg10283362 | GTTATAAGAGAAGAGGTGTGTTGTTTTT | AAACTCACATTACACAACTTAACTCCTTT |
| cg11024728 | TTGTTTTTGGTAGAGAAAGGATAAGA | AAACACAAAACAAATATTCCATACACAC |
| cg11053414 | TTATAGTTGAGGAGATATTGGTTAGAGG | AAATAACTCAATATACATACCTCCCATACTT |
| cg11112615 | TTAGGTTTGATGYGATGAGATTTG | CTCTTTAATAACACATCACAATAAATACAATTACTC |
| cg11543899 | TATGAAGATTTGGTTTGAGTATTGTAAAGAG | TTCACTACAAAATATACTCCCACTCC |
| cg11883129 | GGAAGAATATGGGTTGAATGAGAA | AAATCAAAAACAAAAACCACCTCT |
| cg11968956 | TGGAGTATTATTTGGTTATAAAGAGGAATG | CCCAACCCCTAACAACCAC |
| cg12126686 | TGYGTGTGAAATGTTGGTAGGTG | TTAAATTCTACAACTATTACCCATCTTTC |
| cg12437013 | GTTTTATTTAGTAGTTTTTGAAGGGTTTG | CTACCCACTCTATTCTCRCCCTTAT |
| cg12709880 | GTGTTTTGGTTATATTTGTAGATGATATTG | AAACTACTACAACAACATTTACTACCTCATT |
| cg13388253 | TTTTGGGTTTGAGGGAGGA | CCAAACCTACCCTCCCAAATC |
| cg13860573 | GGGTGTTTGGAAATATTGTTTTGAG | CAATATCTCCACCAACACAACC |
| cg14186948 | GGTTGTTGTATTTTGGTATTAGTTTTAGTT | AACCTCCAAAAACACAACCAC |
| cg14933993 | TTTTGGTGAGTAGAGTATTTTGTGTTG | AAATAACTTCAAAAACACCACAAAAC |
| cg16092154 | GGGGAGAAGGGGTGAGGT | TAAAACCACCCTATCTAACAAACTCAA |
| cg16113883 | GATTTAAAATAGTGAGAGTTGTAAATTATAGGTAAG | CRTAAAATAATCCAAAACCTAAAAATYTAAAC |
| cg16667827 | AGTAYGTTGTGTAAGAGTATATTATTTGTTTAAATG | AAACTCATTTACCCCTATTTACTCTTTC |
| cg17073989 | GTTTGAGTATGAAGTTAGATTAATAGAGATTAAAGA | TATACATACATATCTTAATACCACAAACAACA |
| cg18187326 | GTGGATAAATAGTTGTTTGTTAATGTGGT | ACCCTAACTCAACCAAATAAAATAAAAATC |
| cg18634211 | TGGTTGGATAYGGAGTTTATGGT | AAAAACTACCCRCRAAAAATTAAATCA |
| cg20049730 | GTTTGGATGGGAGGGAGATAGT | AAAATTCCTAACCAAATCATCTCATC |
| cg22082780 | TTTTTGTAAAGGGGAAGTTTTGTTG | AAAAACTTAAAACCCCAACTAACATT |
| cg22110428 | TTGGTGGAAGGAAGTAATGGT | CTCCTCAACAATAAACTATTTCTCTATATCAC |
| cg22275446 | ATAGATTGAATGAGATTATTATTTGGAGAGTT | CTCCTTAACTCATTACTCCTCCACAA |
| cg22282916 | TTTAAGGGATTAATTTTTGTTAGGTGTTT | CCACTTAACACAATTTCCCCTTAC |
| cg23875752 | TGAYGGGGAAGGGTTATGG | ACCCRTAATTTCCCRTTTATTCA |
| cg24419094 | AAAAGAATATTGGAGGGAAAAATTG | AACCCAATCTACCTTCTTCTTAACAC |
| cg25937052 | GAGGTGGAATGTATTATTTGGAGA | CAAAAACATAACCCCTACCCTTA |

**Supplementary Table 3 Distribution of DMPs in chromosomes and genomic regions**

| **Chromosome** | **DMP associated genes** |
| --- | --- |
| Chr1 | *C1orf170, CAMTA1, CASZ1, FBXO28, FLG-AS1, HEATR1, HMCN1, HSPA7, KIAA0040, KLHDC8A, LGALS8, LIN28, LINC00466, LPPR5, LRRC41, LRRC8D, NKAIN1, OR2B11, PADI4, PEX14, PLA2G4A, PPP2R5A, PRDM16, RERE, RUNX3, SHE, SMG7-AS1, SSX2IP* |
| Chr2 | *ACOXL, ADCY3, ARHGAP15, BRE, CHMP3, CRIM1, DNAJC27, FOXN2, GPD2, HTR2B, LINC00607, LOC100130691, LYG2, MIR548N, NCL, PRKCE, RAMP1, RNF103-CHMP3, RRM2, RTN4, SULT1C2, UBR3* |
| Chr3 | *ATP13A3, ATXN7, B4GALT4, CBLB, FHIT, HHLA2, LINC00636, NCK1, SEC22A, SH3BP5, TM4SF1, TMEM108* |
| Chr4 | *BBS7, CCDC109B, DCUN1D4, DTHD1, EXOSC9, FAM47E, FBXW7, LIMCH1, LRPAP1, MAML3, PCGF3, TBC1D1, TBC1D19, TSPAN5* |
| Chr5 | *AHRR, AMACR, BTNL9, CCNH, FAM105A, ITGA1, MYOZ3, RANBP17, RGNEF, SETD9* |
| Chr6 | *C6orf208, C6orf70, DUSP22, ELOVL5, FAM184A, FKBP5, GCNT2, HLA-DPA1, HLA-DPB2, HLA-DRB1, IPCEF1, LINC01268, LOC100132735, LRRC16A, NELFE, NUDT3, PACSIN1, RANBP9, SERPINB6, SLC35B3, SYNE1, TMEM151B, TMEM181, TNFAIP3* |
| Chr7 | *C7orf58, C7orf73, HEPACAM2, HOXA4, LINC-PINT, NRCAM, PL-5283, PRKAR1B* |
| Chr8 | *C8orf37-AS1, C8orf71, CCDC26, GSDMD, LINC00051, LINC00588, TG, MTUS1, MYOM2, NSMCE2, SDC2, SMIM19, VPS28* |
| Chr9 | *C9orf129, C9orf5, COL5A1, HDHD3, JAK2, MIR600, MLLT3* |
| Chr10 | *CALHM1, FAM190B, FAM45A, HELLS, MGMT, OPTN, PDZD7, PTPRE, SGPL1, SLC29A3, TMEM72, USP6NL, VCL* |
| Chr11 | *ETS1, FLJ42102, KCNQ1, MS4A3, NADSYN1, OR51I1, PAMR1, PSMC3, ZNF215* |
| Chr12 | *CHST11, CLEC2D, CLIP1, FAM101A, KIAA1467, KLRC4-KLRK1, PAWR, PWP1, ZNF664* |
| Chr13 | *ARHGEF7, EPSTI1, FARP1, LINC00332, LINC01044, MRP63, TMCO3* |
| Chr14 | *MAX, PELI2, PRPF39, RAD51L1, RPS6KA5, TMEM260, TSHR* |
| Chr15 | *ATP8B4, CORO2B, CYFIP1, HDC, IGF1R, IQGAP1, MTHFS, PHGR1, SMAD6, SNUPN* |
| Chr16 | *ACSF3, MRPL28, MRPS34, RAB11FIP3, TK2* |
| Chr17 | *ABR, BRCA1, GUCY2D, KRT13, KRT40, RPH3AL, SEC14L1, TBC1D16, TMEM104, VMP1* |
| Chr18 | *MBP, MYOM1, NPC1, PSTPIP2* |
| Chr19 | *CD22, CEACAM18, KLK15, KLK8, LOC101929124, LOC284379, PRR12, TNFAIP8L1, ZNF304* |
| Chr20 | *GPCPD1, ITCH, LOC149837, OSBPL2, PLCG1-AS1, PPP1R16B, SPAG4L* |
| Chr21 | *AGPAT3, COL18A1, KRTAP21-1, MIR99AHG, NCAM2* |
| Chr22 | *MTMR3, PACSIN2, PHF21B, TNRC6B, ZNRF3* |

**Supplementary Table 4 Fisher’s exact test of DMP distribution across gene and CpG island-related regions**

| **Region** | **DMP Count** | **DMP Proportion (%)** | **All probe Count** | **All Probe Proportion (%)** | **Odds Ratio** | ***P*_value** | **adj_*P*_value** |
| --- | --- | --- | --- | --- | --- | --- | --- |
| **Gene regions** |  |  |  |  |  |  |  |
| 1stExon | 7 | 1.68 | 21727 | 3 | 0.553069379 | 0.147590448 | 0.295180896 |
| 3'UTR | 6 | 1.44 | 18084 | 2.5 | 0.571117851 | 0.206925091 | 0.331080146 |
| 5'UTR | 39 | 9.38 | 62457 | 8.63 | 1.095426061 | 0.599685586 | 0.690290671 |
| Body | 159 | 38.22 | 271645 | 37.53 | 1.029898691 | 0.761557569 | 0.761557569 |
| ExonBnd | 0 | 0 | 5094 | 0.7 | 0 | 0.128894477 | 0.295180896 |
| IGR | 139 | 33.41 | 199534 | 27.57 | 1.318569228 | 0.008424776 | 0.046847728 |
| TSS1500 | 48 | 11.54 | 90746 | 12.54 | 0.90996823 | 0.604004337 | 0.690290671 |
| TSS200 | 18 | 4.33 | 54551 | 7.54 | 0.554851057 | 0.011711932 | 0.046847728 |
| **CpG island-related regions** |  |  |  |  |  |  |  |
| Island | 38 | 9.13 | 133572 | 18.45 | 0.444084375 | 1.7271E-07 | 1.03626E-06 |
| N_Shelf | 10 | 2.4 | 25813 | 3.57 | 0.665917919 | 0.233852727 | 0.467705454 |
| N_Shore | 42 | 10.1 | 71325 | 9.85 | 1.027386752 | 0.869147991 | 0.89100524 |
| opensea | 283 | 68.03 | 408211 | 56.4 | 1.645664491 | 1.45185E-06 | 4.35554E-06 |
| S_Shelf | 14 | 3.37 | 24021 | 3.32 | 1.014612172 | 0.89100524 | 0.89100524 |
| S_Shore | 29 | 6.97 | 60896 | 8.41 | 0.815679562 | 0.330806572 | 0.496209859 |

**Supplementary Table 5 Fisher’s exact test of hypermethylated DMP distribution across gene and CpG island-related regions**

| **Region** | **Hypermethylated DMP Count** | **Hypermethylated DMP Proportion (%)** | **All probe Count** | **All Probe Proportion (%)** | **Odds Ratio** | ***P*_value** | **adj_*P*_value** |
| --- | --- | --- | --- | --- | --- | --- | --- |
| **Gene regions** |  |  |  |  |  |  |  |
| 1stExon | 2 | 1.32 | 21727 | 3 | 0.433760584 | 0.33586917 | 0.67173834 |
| 3'UTR | 1 | 0.66 | 18084 | 2.5 | 0.260148439 | 0.19241651 | 0.577210905 |
| 5'UTR | 14 | 9.27 | 62457 | 8.63 | 1.082091759 | 0.771103998 | 0.771103998 |
| Body | 53 | 35.1 | 271645 | 37.53 | 0.900254731 | 0.557501765 | 0.721779052 |
| ExonBnd | 0 | 0 | 5094 | 0.7 | 0 | 0.631556671 | 0.721779052 |
| IGR | 53 | 35.1 | 199534 | 27.57 | 1.421070893 | 0.0447137 | 0.3577096 |
| TSS1500 | 21 | 13.91 | 90746 | 12.54 | 1.126951228 | 0.622229457 | 0.721779052 |
| TSS200 | 7 | 4.64 | 54551 | 7.54 | 0.596383414 | 0.21645409 | 0.577210905 |
| **CpG island-related regions** |  |  |  |  |  |  |  |
| Island | 18 | 11.92 | 133572 | 18.45 | 0.598076377 | 0.036052509 | 0.108157526 |
| N_Shelf | 3 | 1.99 | 25813 | 3.57 | 0.548138075 | 0.382881282 | 0.574321923 |
| N_Shore | 17 | 11.26 | 71325 | 9.85 | 1.160622983 | 0.583536724 | 0.583536724 |
| opensea | 101 | 66.89 | 408211 | 56.4 | 1.561851741 | 0.010721174 | 0.064327044 |
| S_Shelf | 3 | 1.99 | 24021 | 3.32 | 0.590543014 | 0.495647297 | 0.583536724 |
| S_Shore | 9 | 5.96 | 60896 | 8.41 | 0.689957717 | 0.376683408 | 0.574321923 |

**Supplementary Table 6 Fisher’s exact test of hypomethylated DMP distribution across gene and CpG island-related regions**

| **Region** | **Hypomethylated DMP Count** | **Hypomethylated DMP Proportion (%)** | **All probe Count** | **All Probe Proportion (%)** | **Odds Ratio** | ***P*_value** | **adj_*P*_value** |
| --- | --- | --- | --- | --- | --- | --- | --- |
| **Gene regions** |  |  |  |  |  |  |  |
| 1stExon | 5 | 1.89 | 21727 | 3 | 0.621443701 | 0.367705899 | 0.546625747 |
| 3'UTR | 5 | 1.89 | 18084 | 2.5 | 0.750507999 | 0.693032289 | 0.693032289 |
| 5'UTR | 25 | 9.43 | 62457 | 8.63 | 1.103037881 | 0.585547099 | 0.669196684 |
| Body | 106 | 40 | 271645 | 37.53 | 1.109761909 | 0.40996931 | 0.546625747 |
| ExonBnd | 0 | 0 | 5094 | 0.7 | 0 | 0.272483128 | 0.546625747 |
| IGR | 86 | 32.45 | 199534 | 27.57 | 1.262470777 | 0.085335793 | 0.341343171 |
| TSS1500 | 27 | 10.19 | 90746 | 12.54 | 0.791457708 | 0.306558949 | 0.546625747 |
| TSS200 | 11 | 4.15 | 54551 | 7.54 | 0.531305787 | 0.0354589 | 0.283671198 |
| **CpG island-related regions** |  |  |  |  |  |  |  |
| Island | 20 | 7.55 | 133572 | 18.45 | 0.360739186 | 6.8713E-07 | 4.12278E-06 |
| N_Shelf | 7 | 2.64 | 25813 | 3.57 | 0.733685466 | 0.508813303 | 0.763219954 |
| N_Shore | 25 | 9.43 | 71325 | 9.85 | 0.952961356 | 0.917888503 | 0.917888503 |
| opensea | 182 | 68.68 | 408211 | 56.4 | 1.695432036 | 5.21354E-05 | 0.000156406 |
| S_Shelf | 11 | 4.15 | 24021 | 3.32 | 1.261686573 | 0.392040828 | 0.763219954 |
| S_Shore | 20 | 7.55 | 60896 | 8.41 | 0.888684887 | 0.73929921 | 0.887159052 |

**Supplementary Table 7 Distribution of DMPs and all probes across chromosomes**

| **Chromosome** | **DMP Count** | **DMP Proportion (%)** | **All probe Count** | **All Probe Proportion (%)** |
| --- | --- | --- | --- | --- |
| 1 | 47 | 11.3 | 70397 | 9.73 |
| 2 | 38 | 9.13 | 55908 | 7.72 |
| 3 | 19 | 4.57 | 42720 | 5.9 |
| 4 | 15 | 3.61 | 31348 | 4.33 |
| 5 | 23 | 5.53 | 38649 | 5.34 |
| 6 | 40 | 9.62 | 45223 | 6.25 |
| 7 | 22 | 5.29 | 38721 | 5.35 |
| 8 | 22 | 5.29 | 32781 | 4.53 |
| 9 | 13 | 3.12 | 22493 | 3.11 |
| 10 | 19 | 4.57 | 35642 | 4.92 |
| 11 | 21 | 5.05 | 42325 | 5.85 |
| 12 | 25 | 6.01 | 38589 | 5.33 |
| 13 | 14 | 3.37 | 18024 | 2.49 |
| 14 | 11 | 2.64 | 25619 | 3.54 |
| 15 | 14 | 3.37 | 24441 | 3.38 |
| 16 | 8 | 1.92 | 32072 | 4.43 |
| 17 | 17 | 4.09 | 38255 | 5.29 |
| 18 | 7 | 1.68 | 13192 | 1.82 |
| 19 | 13 | 3.12 | 32508 | 4.49 |
| 20 | 13 | 3.12 | 20378 | 2.82 |
| 21 | 8 | 1.92 | 8935 | 1.23 |
| 22 | 7 | 1.68 | 15618 | 2.16 |

**Supplementary Table 8 Characteristics of DMRs identified by the Bumphunter algorithm in DCD**

| **Region** | **Chromosome** | **Start** | **End** | **Width** | **UCSC RefGene Name** |
| --- | --- | --- | --- | --- | --- |
| DMR_1 | 1 | 8484417 | 8484703 | 286 | RERE |
| DMR_2 | 1 | 207224090 | 207224102 | 12 | YOD1 |
| DMR_3 | 2 | 113992694 | 113993313 | 619 | PAX8, PAX8-AS1 |
| DMR_4 | 2 | 183943175 | 183943938 | 763 | DUSP19 |
| DMR_5 | 2 | 219157103 | 219157119 | 16 | PNKD |
| DMR_6 | 2 | 26401598 | 26401878 | 280 | FAM59B, GAREML |
| DMR_7 | 2 | 201390294 | 201390305 | 11 | SGOL2 |
| DMR_8 | 2 | 178130312 | 178130568 | 256 | NFE2L2 |
| DMR_9 | 2 | 242051650 | 242052583 | 933 | PASK |
| DMR_10 | 3 | 179169252 | 179169350 | 98 | GNB4 |
| DMR_11 | 3 | 149094653 | 149095283 | 630 | TM4SF1 |
| DMR_12 | 4 | 122721721 | 122721982 | 261 | EXOSC9 |
| DMR_13 | 5 | 56204405 | 56204995 | 590 | SETD9, C5orf35 |
| DMR_14 | 5 | 157079404 | 157079668 | 264 | SOX30 |
| DMR_15 | 5 | 126408756 | 126409372 | 616 | FLJ44606 |
| DMR_16 | 5 | 135415762 | 135416529 | 767 | MIR886, |
| DMR_17 | 6 | 33091567 | 33091841 | 274 | HLA-DPB2 |
| DMR_18 | 6 | 31275148 | 31275881 | 733 |  |
| DMR_19 | 6 | 25041912 | 25042548 | 636 | FAM65B |
| DMR_20 | 6 | 28129416 | 28129656 | 240 | ZNF389, ZNF192P1 |
| DMR_21 | 6 | 42927940 | 42928079 | 139 | GNMT |
| DMR_22 | 6 | 106546540 | 106546824 | 284 | PRDM1 |
| DMR_23 | 7 | 27169957 | 27171051 | 1094 | HOXA4 |
| DMR_24 | 7 | 135346500 | 135346802 | 302 | PL-5283, C7orf73 |
| DMR_25 | 7 | 94953653 | 94954202 | 549 | PON1 |
| DMR_26 | 7 | 120629051 | 120629703 | 652 | C7orf58, CPED1 |
| DMR_27 | 8 | 1765066 | 1765679 | 613 | MIR596 |
| DMR_28 | 9 | 124989052 | 124990276 | 1224 | LHX6 |
| DMR_29 | 11 | 63656088 | 63656342 | 254 | MARK2 |
| DMR_30 | 11 | 58389592 | 58390088 | 496 | CNTF |
| DMR_31 | 12 | 108078821 | 108079287 | 466 | PWP1 |
| DMR_32 | 12 | 132312106 | 132312461 | 355 | MMP17 |
| DMR_33 | 12 | 13153422 | 13153430 | 8 | HTR7P, HEBP1 |
| DMR_34 | 12 | 9821504 | 9822287 | 783 | CLEC2D |
| DMR_35 | 14 | 24779793 | 24780557 | 764 | LTB4R2 |
| DMR_36 | 15 | 80189821 | 80190137 | 316 | MTHFS |
| DMR_37 | 15 | 91473059 | 91473569 | 510 | UNC45A |
| DMR_38 | 15 | 101389272 | 101389974 | 702 |  |
| DMR_39 | 16 | 70473223 | 70473447 | 224 | ST3GAL2 |
| DMR_40 | 16 | 14402939 | 14403425 | 486 | ,MIR365-1, MIR365A |
| DMR_41 | 16 | 419916 | 420255 | 339 | MRPL28 |
| DMR_42 | 16 | 8806966 | 8807308 | 342 | ABAT |
| DMR_43 | 17 | 5402883 | 5404090 | 1207 | LOC728392 |
| DMR_44 | 17 | 800512 | 800761 | 249 | NXN |
| DMR_45 | 17 | 41277730 | 41278563 | 833 | BRCA1 |
| DMR_46 | 19 | 55972791 | 55973338 | 547 | ISOC2 |
| DMR_47 | 19 | 46806907 | 46807272 | 365 | HIF3A |
| DMR_48 | 22 | 46449430 | 46450251 | 821 | C22orf26, PRR34-AS1 |

**Supplementary Table 9 Characteristics of DMRs identified by the ProbeLasso algorithm in DCD**

| **Region** | **Chromosome** | **Start** | **End** | **Width** | **UCSC RefGene Name** |
| --- | --- | --- | --- | --- | --- |
| DMR_1 | 1 | 32715396 | 32718568 | 3173 | FAM167B, LCK |
| DMR_2 | 1 | 154941734 | 154945497 | 3764 | SHC1 |
| DMR_3 | 3 | 52529028 | 52530021 | 994 | STAB1 |
| DMR_4 | 5 | 78985403 | 78986396 | 994 | CMYA5 |
| DMR_5 | 5 | 80528603 | 80529596 | 994 | CKMT2 |
| DMR_6 | 5 | 148809329 | 148810322 | 994 |  |
| DMR_7 | 6 | 30459418 | 30461531 | 2114 | HLA-E |
| DMR_8 | 6 | 31539926 | 31546206 | 6281 | LTA, TNF |
| DMR_9 | 6 | 31758586 | 31762929 | 4344 | VARS |
| DMR_10 | 6 | 33864254 | 33874099 | 9846 | RP3-468B3.2 |
| DMR_11 | 10 | 106093613 | 106097088 | 3476 | ITPRIP |
| DMR_12 | 13 | 31506467 | 31507635 | 1169 | TEX26 |
| DMR_13 | 15 | 31514513 | 31517719 | 3207 | KLF13 |
| DMR_14 | 16 | 4713946 | 4714939 | 994 | MGRN1 |
| DMR_15 | 16 | 8806268 | 8807275 | 1008 | ABAT |
| DMR_16 | 16 | 14402168 | 14404663 | 2496 | MIR365A |
| DMR_17 | 16 | 69966567 | 69967560 | 994 | WWP2, MIR140 |
| DMR_18 | 17 | 799766 | 801008 | 1243 | NXN |
| DMR_19 | 17 | 41924259 | 41924840 | 582 | CD300LG |
| DMR_20 | 17 | 75315222 | 75315930 | 709 | SEPT9 |
| DMR_21 | 20 | 5484648 | 5485641 | 994 | LINC00654 |
| DMR_22 | 22 | 42827628 | 42828621 | 994 | NFAM1 |
